# Zonisamide Attenuates α-Synuclein Neurotoxicity by an Aggregation-Independent Mechanism in a Rat Model of Familial Parkinson’s Disease

**DOI:** 10.1371/journal.pone.0089076

**Published:** 2014-02-20

**Authors:** Shigeki Arawaka, Shingo Fukushima, Hiroyasu Sato, Asuka Sasaki, Kaori Koga, Shingo Koyama, Takeo Kato

**Affiliations:** Department of Neurology, Hematology, Metabolism, Endocrinology and Diabetology, Yamagata University Faculty of Medicine, Yamagata, Japan; Hertie Institute for Clinical Brain Research and German Center for Neurodegenerative Diseases, Germany

## Abstract

The anti-epileptic agent zonisamide (ZNS) has been shown to exert protective effects in neurotoxin-based mouse models of Parkinson disease. However, it is unknown whether ZNS can attenuate toxicity of familial Parkinson’s disease-causing gene products. In this study, we investigated the effects of ZNS on neurodegeneration induced by expression of A53T α-synuclein in the rat substantia nigra using a recombinant adeno-associated virus vector. Expression of A53T α-synuclein yielded severe loss of nigral dopamine neurons and striatal dopamine nerve terminals from 2 weeks to 4 weeks after viral injection. Oral administration of ZNS (40 mg/kg/day) significantly delayed the pace of degeneration at 4 weeks after viral injection as compared with the vehicle group. This effect lasted until 8 weeks after viral injection, the final point of observation. ZNS treatment had no impact on the survival of nigrostriatal dopamine neurons in rats expressing green fluorescent protein. Quantification of striatal Ser129-phosphorylated α-synuclein-positive aggregates showed that these aggregates rapidly formed from 2 weeks to 4 weeks after viral injection. This increase was closely correlated with loss of nigrostriatal dopamine neurons. However, ZNS treatment failed to alter the number of all striatal Ser129-phosphorylated α-synuclein-positive aggregates, including small dot-like and large round structures. The number of these aggregates was almost constant at 4 weeks and 8 weeks after viral injection, although ZNS persistently prevented loss of nigrostriatal dopamine neurons during this period. Also, ZNS treatment did not affect the number of striatal aggregates larger than 10 µm in diameter. These data show that ZNS attenuates α-synuclein-induced toxicity in a manner that is independent of the formation and maturation of α-synuclein aggregates in an *in vivo* model of familial Parkinson’s disease, suggesting that ZNS may protect nigrostriatal dopamine neurons by modulating cellular damage or a cell death pathway commonly caused by neurotoxins and α-synuclein.

## Introduction

Parkinson’s disease is a common neurodegenerative disorder characterized clinically by bradykinesia, rigidity, tremor, and gait dysfunction [1]. In the treatment of Parkinson’s disease, levodopa is used as the ‘gold standard’, because it dramatically improves these motor impairments and allows patients to engage in independent activities and remain employable for a longer time than they would not receive the treatment [2]. In addition, levodopa is known to increase survival rates [1]. For most patients, levodopa provides satisfactory disease control in early stages of Parkinson’s disease. However, Parkinson’s disease patients eventually suffer deterioration of motor abilities and often experience adverse events from levodopa, such as fluctuating motor responses, dyskinesia, and psychiatric problems [3]. Quality of life is further reduced by the appearance of symptoms that do not respond to levodopa, including postural instability, autonomic dysfunction, and dementia [3]. Levodopa is the most potent symptomatic treatment against motor impairments by a dopamine replacement, but it is unable to prevent disease progression. An urgent need for Parkinson’s disease therapy is to find a neuroprotective treatment that slows or stops progression of the disease [4]. There are many candidate agents showing neuroprotective effects in laboratory models using the neurotoxins, 1-methyl-4-phenyl-1,2,3,6-tetrahydropyrydine (MPTP) and 6-hydroxy dopamine (6-OHDA) [5], [6]. However, none have been proven to have disease-modifying effects in patients with Parkinson’s disease [4]. As one of the reasons for this disconnect, there is difficulty in discriminating the neuroprotective effects of candidate agents from their symptomatic effects by elevation of synaptic dopamine levels or stimulation of dopamine receptors in clinical trials [4]. In addition, it is unclear whether neurotoxin-based animal models precisely reflect the pathogenesis of Parkinson’s disease [4], [7]. There has been little study to clarify whether candidate agents can protect against neurodegeneration caused by familial Parkinson’s disease-linked gene products in genetic animal models.

Zonisamide (1,2-benzisoxazole-3-methanesulfonamide: ZNS) was originally synthesized in Japan and it is clinically used as an anti-epileptic agent in Japan, South Korea, the United States, and Europe. Although the exact mechanism remains to be elucidated, ZNS is proposed to act against epilepsy through inhibition of voltage-dependent Na^+^ and T-type Ca^2+^ channels. In addition, a Japanese group revealed that the combination of low-dose ZNS (25–100 mg/day) and levodopa improved motor impairments in patients with Parkinson’s disease in a randomized, double-blind, and placebo-controlled study [8]. This discovery has promoted studies to find the pharmacological effects of ZNS other than ion channel inhibition. An earlier article reported that acute administration of ZNS (20 and 50 mg/kg) in rats increased intracellular and extracellular levels of dopamine and its metabolites in the striatum through inhibition of monoamine oxidase type B (MAO-B) activity [9]. Alternatively, ZNS has been reported to increase the activity and expression of tyrosine hydroxylase (TH) [10], and stimulate dopamine release in the rat striatum [9]. ZNS may improve motor impairments of Parkinson’s disease by its multiple effects on dopamine release and synthesis [11]. Furthermore, experiments using neurotoxin-based mouse models have provided insights into the protective effects of ZNS against neuronal death. In MPTP-treated mice, intraperitoneal administration of ZNS (20 mg/kg) mitigated loss of nigral TH-positive neurons with attenuating dopamine depletion in the striatum [10]. In mice that received 6-OHDA to induce hemiparkinsonism, repeated intraperitoneal injection of ZNS (30 mg/kg) for 7 days also abrogated the reduction in nigral dopamine neurons [12]. This protective effect of ZNS was proposed to enhance the cysteine transport system in astrocytes, resulting in induction of the synthesis of glutathione, which has anti-oxidative activity. Accumulating data show that ZNS has protective effects in neurotoxin-based mouse models [10]–[14]. In this study, we tested whether ZNS protects against neurotoxicity induced by familial Parkinson’s disease-linked A53T α-synuclein in a recombinant adeno-associated virus (rAAV)-based rat model. We report here that ZNS exerts protective effects against A53T α-synuclein-induced neurodegeneration in a manner that is independent of α-synuclein aggregation in this rat model.

## Materials and Methods

### Ethics Statement

The experiments using rats had been approved by the Animal Subjects Committee of Yamagata University.

### Preparation of Recombinant Adeno-associated Viral Vectors

We prepared rAAV particles using the AAV Helper-Free system (Stratagen) as described previously [15]. This AAV transduction system consists of pAAV expression, pAAV-RC packaging (it contains AAV2 rep and cap genes), and pAAV helper plasmids. The original expression plasmid, pAAV-MCS, contains the inverted terminal repeats of AAV2 and a cytomegalovirus promoter-driven cassette for the expression of the target gene product. We replaced this cassette with the compound cytomegalovirus enhancer/chicken β-actin (CAG) promoter [16], and added the woodchuck hepatitis virus post-transcriptional regulatory element (WPRE) downstream of a multiple cloning site to enhance the target gene expression [17]. As the target gene of interest, we inserted human A53T α-synuclein and GFP into the cloning site, which were termed rAAV-A53T α-synuclein and rAAV-GFP, respectively. For rAAV particle production, HEK293 derived cells (AAV-293 cells, which stably express the adenovirus E1 gene to effectively produce viral particles in the AAV Helper-Free system, Stratagen) were grown to 80% confluence in 225-cm^2^ flasks, and they were co-transfected with three plasmids using Lipofectamine LTX (Life Technologies). After incubation for 3 days, the cell pellet was collected, suspended in the lysis solution (50 mM Tris, pH 8.0, 150 mM NaCl), and lysed by repeating three freeze/thaw cycles. Lysates were treated with 50 U/ml benzonase (Sigma) and centrifuged to remove cellular debris. The resultant supernatant was purified by centrifugation (302,000×g for 2 h at 18°C) on discontinuous iodixanol gradients (Optiprep; Axis-Shield). The fractions containing viral particles were further purified by heparin-agarose column chromatography (Sigma) [18]. After treating the viral solutions with DNase I and proteinase K (Roche) in turn, the viral genome copy number was determined by quantitative real-time PCR using primers for the WPRE sequence.

### Animals and Stereotaxic Surgery for Viral Injection

Nine-week-old male Sprague-Dawley rats were purchased from Charles River. Rats were individually housed in cages with ad libitum access to food and water under 12-h light/dark cycle conditions. The experiments had been approved by the Animal Subjects Committee of Yamagata University. Before surgery, rats were anesthetized by pentobarbital, and they were placed in a stereotaxic frame (Narishige) [15]. All injections were made unilaterally into the left substantia nigra with the head placed in a flat-skull position at 5.2 mm posterior and 2.0 mm left to bregma and 7.7 mm ventral to dura, according to the rat brain atlas of Paxinos and Watson [19]. 4 µl of viral solutions containing 1.36×10^9^ genome copies were injected with a 22-gauge needle and a 25-µl Hamilton syringe at a speed of 250 nl/min using a microsyringe pump (World Precision Instruments). After viral injection, the needle was kept in place for 10 min before withdrawal.

### Zonisamide Treatment

ZNS was provided by Dainippon Sumitomo Pharma. ZNS was suspended in a 0.5% tragacanth gum (Wako) solution before use. We administrated the ZNS solution at a dose of 40 mg/kg by oral gavage once daily (ZNS group). We continued the administration of ZNS until the day before sacrifice as described later. As a control, the same volume of vehicle was simultaneously administrated in a separate set of subjects (vehicle group). Unless otherwise stated, ZNS administration was started 3 days before the viral injection.

### Immunohistochemistry

Under diethyl ether anesthesia, animals were transcardially perfused with physiological saline followed by 4% paraformaldehyde (PFA)/phosphate-buffered saline (PBS) (10 mM phosphate, 137 mM NaCl, 2.7 mM KCl) [15]. Their brains were removed and post-fixed in 8% PFA/PBS containing 4% sucrose for 48 h. The brains were equilibrated stepwise with 7.5%, 15%, and 30% sucrose. These brains were coronally sectioned on a freezing microtome at a thickness of 30 µm. Sections were collected in ten series to be regularly spaced at intervals of 300 µm from each other. Immunohistochemical staining was performed on free-floating sections. Sections were permeabilized with 0.1% Triton X-100/PBS for 10 min and treated with 0.3% hydrogen peroxide for 5 min. After blocking in 3% normal goat serum for 30 min, the sections were incubated with the primary antibody with gentle shaking at 4°C overnight. After washing, the sections were incubated with the appropriate biotinylated secondary antibody for 1 h, followed by avidin-biotin-peroxidase complex (Vector Laboratories) for 1 h. The sections were visualized using 3,3-diaminobenzidine. The following primary antibodies were used: anti-tyrosine hydroxylase (TH; AB152, 1: 2000; Millipore), anti-dopamine transporter (DAT; sc-1433, 1: 2000; Santa Cruz Biotechnology), anti-human α-synuclein (LB509, 1: 200; Zymed Laboratories), and anti-Ser129-phosphorylated α-synuclein antibodies (EYPSYN-01, 1: 200; courtesy of Eisai) [15].

### Counting of Nigral Cell Number and Measurement of Striatal Optical Density

To assess the total number of nigral dopamine neurons, we used sections covering the entire substantia nigra from caudal (bregma ∼ −4.60 mm) to rostral (bregma ∼ −6.60 mm) boundaries according to the rat brain atlas. This yielded six to seven serial sections for each animal. After staining these sections with antibody against TH or DAT, an unbiased stereological estimation was performed by an optical fractionator method using Stereo Investigator software (MicroBrightField). The region of interest was traced and sampled using an Olympus BX50 microscope at magnifications of 4× and 20×, respectively. The counting parameters were the x-y sampling grid size (180×180 µm), the counting frame size (160×160 µm), the dissector height (6 µm), and the guard-zone thickness (3 µm). The obtained counts were verified to archive a Gunderson’s coefficient of error less than 0.10.

We measured striatal dopamine nerve terminals by semiquantifing their optical density [15]. After staining sections with antibody against TH or DAT, the nearest section to bregma was determined according to the rat brain atlas. We analyzed nine sections from ∼ +1.2 mm to ∼ –1.2 mm relative to bregma. The optical densities of immunoreactive fibers were measured using Image J software (version 1.45 s, National Institutes of Health). Nonspecific background intensities were simultaneously assessed by measuring the optical densities of cerebral cortical areas, and subtracted from the total values.

### Counting of Ser129-phosphorylated α-synuclein-positive Aggregate Number

For assessment of α-synuclein aggregates, we counted the number of Ser129-phosphorylated α-synuclein-positive aggregates in the striatum. After staining sections with an antibody for Ser129-phosphorylated α-synuclein, we analyzed the injected sides of three sections, including +0.3 mm, 0 mm, and –0.3 mm relative to bregma, by the optical fractionator method using Stereo Investigator software. The region of interest was traced and sampled using an Olympus BX50 microscope at a magnification of 4× and 10×, respectively. By setting the x-y sampling grid size equal to the counting frame size (330×330 µm), we scanned the whole area of the striatum on the section. We first counted the number of all types of Ser129-phosphorylated α-synuclein-positive aggregates, including small dot-like and large round structures. These aggregates were morphologically defined as axonal swellings and dystrophic terminals. Then, we counted the number of Ser129-phosphorylated α-synuclein-positive aggregates larger than 10 µm in diameter. Those large aggregates were selected by measuring the maximum diameter with a “quick measure circle” tool in this software.

### Western Blotting

At indicated weeks after injection, we cut the brains into 3-mm-thick coronal slices with razor blades using a rat brain slicer matrix (Brain Science Idea). We then selected the slices, including the midbrain or striatum. Furthermore, the midbrains were cut horizontally at the aqueduct, and ventral midbrains were stored at −80°C until use. Tissues were homogenized in 5 vol of buffer [25 mM Tris-HCl, pH 7.4, 137 mM NaCl, 2.7 mM KCl, 2 mM EDTA, 50 mM NaF, 1 mM Na_3_VO_4_, 1 × Protease inhibitor cocktail (Roche), 1 × PhosSTOP (Roche), and 10% glycerol] containing 1% Triton X-100. After 5 min of centrifugation at 1,000×g, the supernatant was collected and ultracentrifuged at 100,000×g for 60 min at 4°C. The resultant supernatant was collected and used for analysis. Samples were denatured by boiling for 5 min in a Laemmli’s sample buffer containing 0.2 M dithiothreitol. To detect DAT, samples were incubated at 37°C for 30 min in the same Laemmli’s sample buffer. Equal amounts of denatured samples were subjected to 12.5% polyacrylamide gel. Western blotting was performed as described previously [20]. To enhance the signals of α-synuclein, the transferred PVDF membrane was incubated in PBS containing 4% PFA for 30 min at room temperature [21], [22]. After washing three times with Tris-buffered saline containing 0.05% Tween 20 (TBS-T), the membrane was blocked in 5% skim milk/TBS-T. In detection of phosphorylated α-syn, the membrane was incubated in buffer containing 50 mM NaF. Signals were detected using a CCD camera, VersaDog 5000 (Bio-Rad). For estimating the expression levels of total or Ser129-phosphorylated α-synuclein, we quantified band intensities of samples using Quantity One software (Bio-Rad). The following primary antibodies were used: anti-TH (AB152, 1: 5000), anti-DAT (sc-1433, 1: 2500), anti-β-actin (AC-15, 1: 10,000; Sigma), anti-total α-synuclein (Syn-1, 1: 5000; BD Biosciences), human α-synuclein (LB509, 1: 5000), and anti-Ser129-phosphorylated α-synuclein (EP1536Y, 1: 5000; Abcam) antibodies.

### Statistical Analysis

The nigral cell number and striatal optical density were expressed as a percentage of the uninjected control side. All values in this study were shown as the mean ± SD. The data were analyzed using a two-way analysis of variance (ANOVA) (vehicle and ZNS groups × observation times), followed by unpaired *t* test for comparisons of only two groups or a Bonferroni’s *post hoc* test as appropriate (SPSS Inc.). Also, comparisons between two groups at one time point were performed by unpaired *t* test (SPSS Inc.). The significance level was set at *P*<0.05.

## Results

### rAAV-mediated Expression of A53T α-synuclein in the Rat Nigrostriatal Neurons

**Figure 1:**
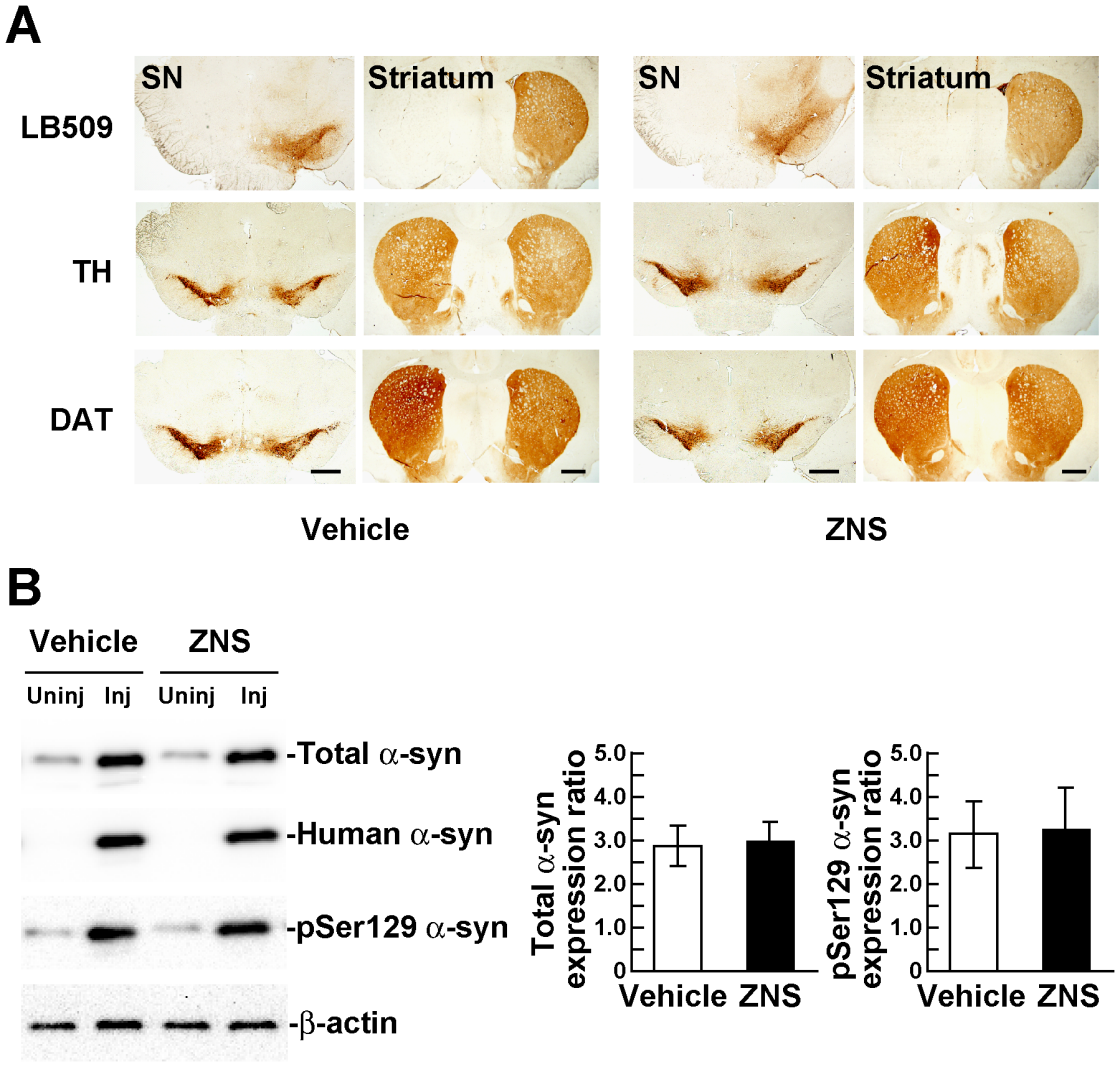
rAAV-mediated expression of A53T α-synuclein in the rat nigrostriatal system. (**A**) rAAV-A53T α-synuclein was unilaterally injected in the substantia nigra pars compacta. Immunohistochemical images of the rat substantia nigra (SN) and striatum at 2 weeks after viral injection. Upper panels show sections immunostained with LB509 antibody that recognizes total human α-synuclein, including phosphorylated and nonphosphorylated forms. Middle and bottom panels show sections immunostained with antibodies for tyrosine hydroxylase (TH) and dopamine transporter (DAT), respectively. Left and right panels show sections of rats treated with vehicle and zonisamide (ZNS), respectively. Scale bars, 1 mm. (**B**) Left panels shows western blotting of the rat ventral midbrains at 2 weeks after viral injection. 1% Triton X-100-soluble extracts (5 µg/lane) were analyzed by western blotting with Syn-1 (total α-synuclein), LB509 (human total α-synuclein), and EP1536Y (phosphorylated α-synuclein) antibodies. For loading control, the same amounts of samples were blotted with β-actin antibody. Right graphs show the ratios of the total α-synuclein and phosphorylated α-synuclein expression levels in the ZNS group relative to those in the vehicle group. Band intensities were normalized to β-actin to correct loading variations between lanes. Data represent the means ± SD.

In this study, we unilaterally injected a rAAV2 vector in the rat substantia nigra to express human A53T α-synuclein, and assessed loss of nigrostriatal dopamine neurons using immunohistochemistry. We first investigated the expression of A53T α-synuclein at 2 weeks after viral injection. Immunohistochemistry with a human α-synuclein specific antibody, LB509, showed that A53T α-synuclein was expressed only on the injected side (Figure 1A). When we compared the staining patterns of A53T α-synuclein with those of TH, which is a maker for dopamine neurons, A53T α-synuclein was expressed mainly in the substantia nigra and to a lesser extent in the surrounding area, including the ventral tegmental area (Figure 1A). A53T α-synuclein was also distributed throughout the injected side of the striatum (Figure 1A). Previous report showed that overexpression of α-synuclein reduced the expression of TH in a MES23.5 dopaminergic cell line [23]. In contrast, treatment with ZNS is shown to elevate the expression of TH in the mouse striatum [10]. To test the validity of immunohistochemistry with anti-TH antibody for the assessment of α-synuclein-induced nigrostriatal degeneration, we also stained the sections with an antibody for the dopamine neuron-specific molecule dopamine transporter (DAT) in most experiments of this study [15]. Representative photographs of these immunostainings of vehicle and ZNS groups at 2 weeks after viral injection were shown in Figure 1A. Quantitative analysis of TH- and DAT-positive nigral neuron number and striatal fiber density is presented in a later section. Next, we biochemically examined the expression levels of α-synuclein in the rat ventral midbrains at 2 weeks after rAAV-A53T α-synuclein injection. In the vehicle group, western blotting with Syn-1 antibody showed that the expression levels of total α-synuclein, including phosphorylated and non-phosphorylated forms, in the injected side were increased 2.88±0.45-fold, as compared with those in the uninjected side (Figure 1B). In the ZNS groups, the expression levels of total α-synuclein in the injected side showed a 2.97±0.46-fold increase to the uninjected side (Figure 1B). The expression levels of Ser129-phosphorylated α-synuclein in the injected sides showed 3.13±0.77-fold and 3.25±0.96-fold increases to the uninjected sides in the vehicle and ZNS groups, respectively (Figure 1B). These findings demonstrated that the expression levels of total and phosphorylated α-synuclein were comparable between the vehicle and ZNS groups at 2 weeks after viral injection (n = 4 animals for each group, unpaired *t* test, P>0.05), indicating that ZNS did not affect gene-transduction efficacy and phosphorylation of α-synuclein at Ser129.

### Effects of Zonisamide on A53T α-synuclein-induced Loss of Nigral Dopamine Neurons

In humans, once-daily oral administration of ZNS at a dose of 50 mg/day elevates the steady-state plasma concentration by 3.5 µg/ml [12]. In mice, when intraperitoneal administration of ZNS is repeated at a dose of 30 mg/kg/day for 14 days, its plasma concentration at 2 h after the final administration is 14.9±0.7 µg/ml, whereas that at 24 h is substantially lower and less than 1 µg/ml [12]. In rats, the plasma concentration of ZNS is also decreased to approximately 2–2.4 µg/ml at 24 h after repeated administration of ZNS (20 mg/kg/day) for 14 days [12]. This is because of the remarkable difference in the half-life of ZNS between rodents (half-life = 8 h) and humans (half-life = 62 h) [12]. In this study, to clarify whether or not ZNS has protective effects against α-synuclein-induced toxicity, we treated rats with ZNS at a higher dose (40 mg/kg/day) to maintain the effective plasma concentration for a longer time. The selected dose is also known to be efficient in a toxin-based mouse model of Parkinson’s disease [14]. In addition, to elicit the effects of ZNS quickly and sufficiently against α-synuclein-induced toxicity as initial assessment, we started administration of ZNS 3 days before viral injection.

**Figure 2:**
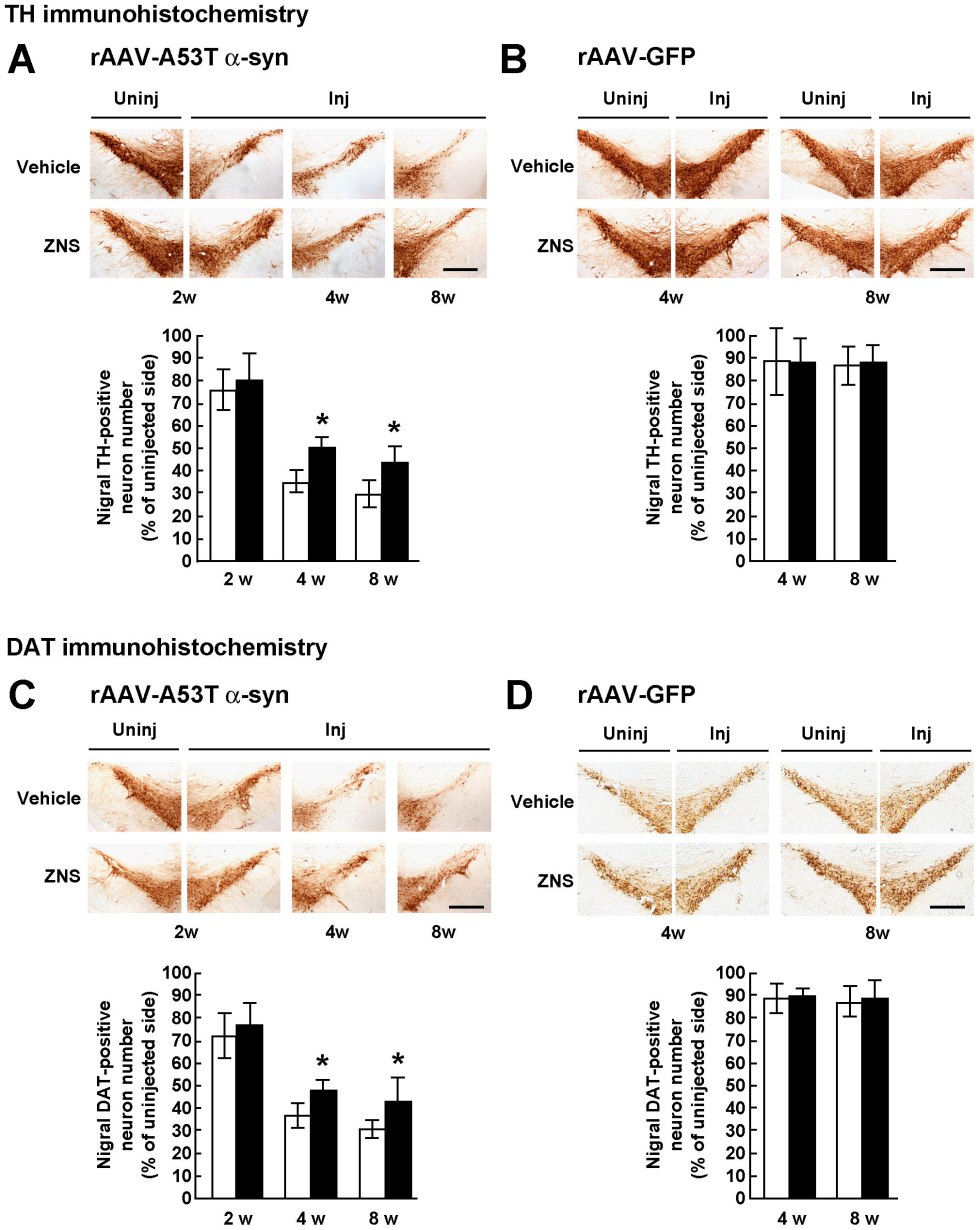
Stereological assessments of the number of nigral dopamine neurons in rats treated with vehicle or ZNS. Rats were unilaterally injected with rAAV-A53T α-synuclein (**A**, **C**) or rAAV-GFP (**B**, **D**). The sections were immunostained with antibody for TH (**A**, **B**) or DAT (**C**, **D**). In rats injected with rAAV-A53T α-synuclein (**A**, **C**), photomicrographs show representative immunostainings of the injected and uninjected sides of the substantia nigra at 2 weeks after injection (left panels), and those of the injected sides at 4 weeks (middle panels) and 8 weeks (right panels) after injection. In rats injected with rAAV-GFP (**B**, **D**), photomicrographs show representative immunostainings of the injected and uninjected sides of the substantia nigra at 4 weeks (left panels) and 8 weeks (right panels) after injection. Scale bars, 0.5 mm. (**A–D**) Graphs show quantitative analysis of TH or DAT-positive nigral neurons. As compared with the vehicle group, ZNS treatment significantly delayed loss of TH-positive nigral neurons at 4 weeks (*P* = 0.004) and 8 weeks (*P* = 0.010) after rAAV-A53T α-synuclein injection. Loss of DAT-positive nigral neurons in the ZNS group was also delayed at 4 weeks (*P* = 0.008) and 8 weeks (*P* = 0.040) after viral injection. The open bars illustrate the vehicle group and the black bars illustrate the ZNS group. Data represent the mean ± SD and *P*-values were estimated by two-way ANOVA, followed by unpaired *t* test (*P<0.05).

To assess loss of nigral dopamine neurons, we stereologically counted TH-positive neurons in the substantia nigra pars compacta. Surviving neurons on the injected side were expressed as a percentage of the total number of neurons on the uninjected side. In the vehicle group, injections of rAAV-A53T α-synuclein yielded drastic loss of nigral neurons at 4 weeks (36.8±6.0%, n = 6) compared with that at 2 weeks (77.6±9.0%, n = 6) after viral injection (Figure 2A). Surviving TH-positive neurons at 8 weeks after viral injection was 29.7±8.4% (n = 6) (Figure 2A). These findings showed that loss of nigral dopamine neurons in this model almost peaked by 4 weeks after viral injection. Two-way ANOVA (group × time) demonstrated a significant main effect of group [*F*(1,30) = 13.766, *P* = 0.001] and a significant main effect of time [*F*(2,30) = 93.178, *P*<0.001]. However, it showed no significant group × time interaction [*F*(2,30) = 1.823, *P* = 0.179]. At 2 weeks after viral injection, there was no difference in the number of TH-positive neurons between the vehicle (77.6±9.0%, n = 6) and ZNS (80.3±11.0%, n = 6) groups (Figure 2A). However, at 4 weeks after viral injection, ZNS treatment significantly delayed loss of TH-positive neurons (50.1±6.2%, n = 6, P = 0.004 by *t* test) (Figure 2A). The protective effect of ZNS lasted until 8 weeks after viral injection, the final point of observation (43.8±6.9%, n = 6, P = 0.010 by *t* test) (Figure 2A).

To clarify α-synuclein-induced neurotoxicity in this model, we then assessed change of neurons by rAAV-mediated gene transfer, including needle insertion, viral infection, and protein overexpression. We further examined whether or not the effects of ZNS involve protection of neurons from this change, because ZNS is reported to prevent neuronal death by scavenging free radicals, such as hydroxyl or nitric oxide, and stabilizing neuronal membranes [24]. We treated rAAV-GFP-injected rats with vehicle or ZNS. In the vehicle group, expression of GFP did not show progressive loss of TH-positive nigral neurons at 8 weeks (86.6±8.2%, n = 7) compared with that at 4 weeks (88.5±14.5%, n = 6) after viral injection (Figure 2B). ZNS treatment did not alter the number of TH-positive neurons at 8 weeks (88.3±7.4%, n = 7) compared with that at 4 weeks (88.4±10.5%, n = 7) after viral injection, indicating that ZNS exerted protective effects against A53T α-synuclein-induced toxicity (Figure 2B).

As mentioned above, overexpression of α-synuclein is reported to reduce the expression of TH in a MES23.5 dopaminergic cell line [23], whereas ZNS is shown to elevate the expression of TH in the mouse striatum [10]. To further assess the effects of ZNS on A53T α-synuclein-induced degeneration of nigral dopamine neurons, we quantified the number of DAT-positive nigral neurons. In the vehicle group at 2 weeks after rAAV-A53T α-synuclein injection, TH-positive nigral neurons of the injected side were 77.6±9.0% of the uninjected side (n = 6) (Figure 2A), while DAT-positive nigral neurons of the injected side were 72.7±10.3% of the uninjected side (Figure 2C). There was no obvious difference in the nigral neuron number between TH and DAT immunostainings. Also, in the ZNS group, TH- and DAT-positive nigral neurons of the injected sides were 80.3±11.0% (n = 6) and 77.0±10.4% (n = 6) of the uninjected sides, respectively (Figure 2A, C). This finding indicated that ZNS did not affect the TH protein levels in our model. Consistent with the results of TH-positive nigral neurons, two-way ANOVA (group × time) demonstrated a significant main effect of group [*F*(1,30) = 11.073, *P* = 0.002] and a significant main effect of time [*F*(2,30) = 72.848, *P*<0.001]. However, it showed no significant group × time interaction [*F*(2,30) = 0.824, *P* = 0.448]. Loss of DAT-positive neurons in the ZNS group was significantly delayed at 4 weeks after viral injection (48.1±4.7%, n = 6), as compared with that in the vehicle group (37.3±6.5%, n = 6, *P* = 0.008 by *t* test) (Figure 2C). At 8 weeks after viral injection, DAT-positive neurons in the ZNS group (43.3±10.5%, n = 6) were more abundant than those in the vehicle group (30.9±4.7%, n = 6, *P* = 0.040 by *t* test) (Figure 2C). In rAAV-GFP-injected rats, there were no significant differences in the number of DAT-positive neurons between the vehicle and ZNS groups at 4 and 8 weeks after viral injection (Figure 2D).

### Effects of Zonisamide on A53T α-synuclein-induced Loss of Striatal Dopamine Innervation

**Figure 3:**
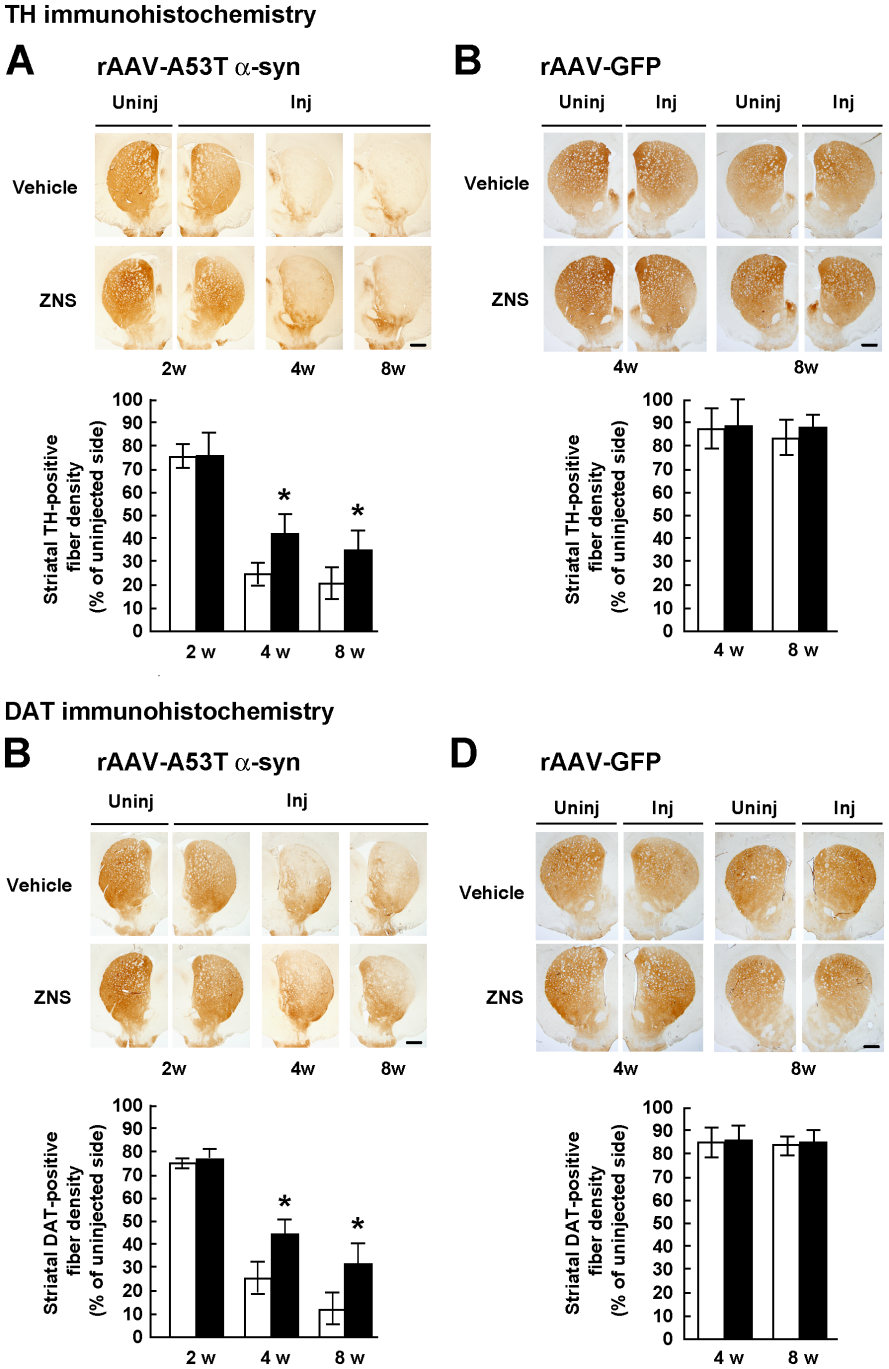
Semiquantification of the optical densities of striatal dopamine nerve terminals in rats treated with vehicle or ZNS. Rats were unilaterally injected with rAAV-A53T α-synuclein (**A**, **C**) or rAAV-GFP (**B**, **D**). The sections were immunostained with antibody for TH (**A**, **B**) or DAT (**C**, **D**). In rats injected with rAAV-A53T α-synuclein (**A**, **C**), photomicrographs show representative immunostainings of the injected and uninjected sides of the substantia nigra at 2 weeks after injection (left panels), and those of the injected sides at 4 weeks (middle panels) and 8 weeks (right panels) after injection. In rats injected with rAAV-GFP (**B**, **D**), photomicrographs show representative immunostainings of the injected and uninjected sides of the substantia nigra at 4 weeks (left panels) and 8 weeks (right panels) after injection. Scale bars, 1.0 mm. (**A–D**) Graphs show semiquantitative analysis of striatal TH or DAT-positive fibers. As compared with the vehicle group, ZNS significantly suppressed loss of striatal TH-positive fibers at 4 weeks (*P*<0.001) and 8 weeks (*P* = 0.004) after rAAV-A53T α-synuclein injection. Decreased densities of DAT-positive fibers were significantly suppressed in the ZNS group at 4 weeks (*P*<0.001) and 8 weeks (*P*<0.001) after viral injection. The open bars illustrate the vehicle group and the black bars illustrate the ZNS group. Data represent the mean ± SD and *P*-values were estimated by two-way ANOVA, followed by a Bonferroni’s *post hoc* test (*P<0.05).

To confirm the protective effects of ZNS on nigral dopamine neurons, we semiquantified the optical densities of TH-positive fibers in the striatum. In the vehicle group, injections of rAAV-A53T α-synuclein remarkably decreased the densities of TH-positive fibers at 4 weeks (26.4±5.5%, n = 6) compared with those at 2 weeks (76.8±4.6%, n = 6) after viral injection (Figure 3A). The densities of TH-positive fibers at 8 weeks after viral injection were 21.8±6.7% (n = 6) (Figure 3A). Two-way ANOVA (group × time) demonstrated a significant main effect of group [*F*(1,30) = 17.210, *P*<0.001], a significant main effect of time [*F*(2,30) = 170.728, *P*<0.001], and a significant group × time interaction [*F*(2,30) = 4.113, *P* = 0.026]. The data were then analyzed by a Bonferroni’s *post hoc* test. At 2 weeks after viral injection, there was no difference in the densities of TH-positive fibers between the vehicle (76.8±4.6%, n = 6) and ZNS (77.2±9.0%, n = 6) groups (*P* = 0.921) (Figure 3A). However, ZNS treatment significantly suppressed loss of striatal fiber innervation at 4 weeks (42.5±7.3%, n = 6, *P*<0.001) and 8 weeks (34.5±8.1%, n = 6, *P* = 0.004) after viral injection (Figure 3A). In rAAV-GFP-injected rats, the vehicle group did not show progressive loss of TH-positive striatal fibers at 8 weeks (84.0±7.2%, n = 7) compared with that at 4 weeks (87.6±8.1%, n = 6) after viral injection (Figure 3B). Also, ZNS treatment did not affect the number of TH-positive striatal fibers at 4 weeks (89.0±11.1%, n = 7) and 8 weeks (88.3±4.8%, n = 7) after viral injection (Figure 3B).

In rAAV-A53T α-synuclein-injected rats, we then analyzed the densities of DAT-positive striatal fibers. Two-way ANOVA (group × time) demonstrated a significant main effect of group [*F*(1,30) = 42.571, *P*<0.001], a significant main effect of time [*F*(2,30) = 237.301, *P*<0.001], and a significant group × time interaction [*F*(2,30) = 6.257, *P* = 0.005]. Decreased densities of DAT-positive striatal fibers were significantly suppressed in the ZNS group (46.1±5.7%, n = 6), as compared with the vehicle group (24.9±7.8%, n = 6) at 4 weeks after viral injection (*P*<0.001) (Figure 3C). At 8 weeks after viral injection, the densities of DAT-positive striatal fibers in the ZNS group (30.3±10.6%, n = 6) were also higher than those in the vehicle group (13.9±6.5%, n = 6, *P*<0.001) (Figure 3C). In rAAV-GFP-injected rats, there were no significant differences in the densities of DAT-positive striatal fibers between the vehicle and ZNS groups at 4 and 8 weeks after viral injection (Figure 3D).

**Figure 4:**
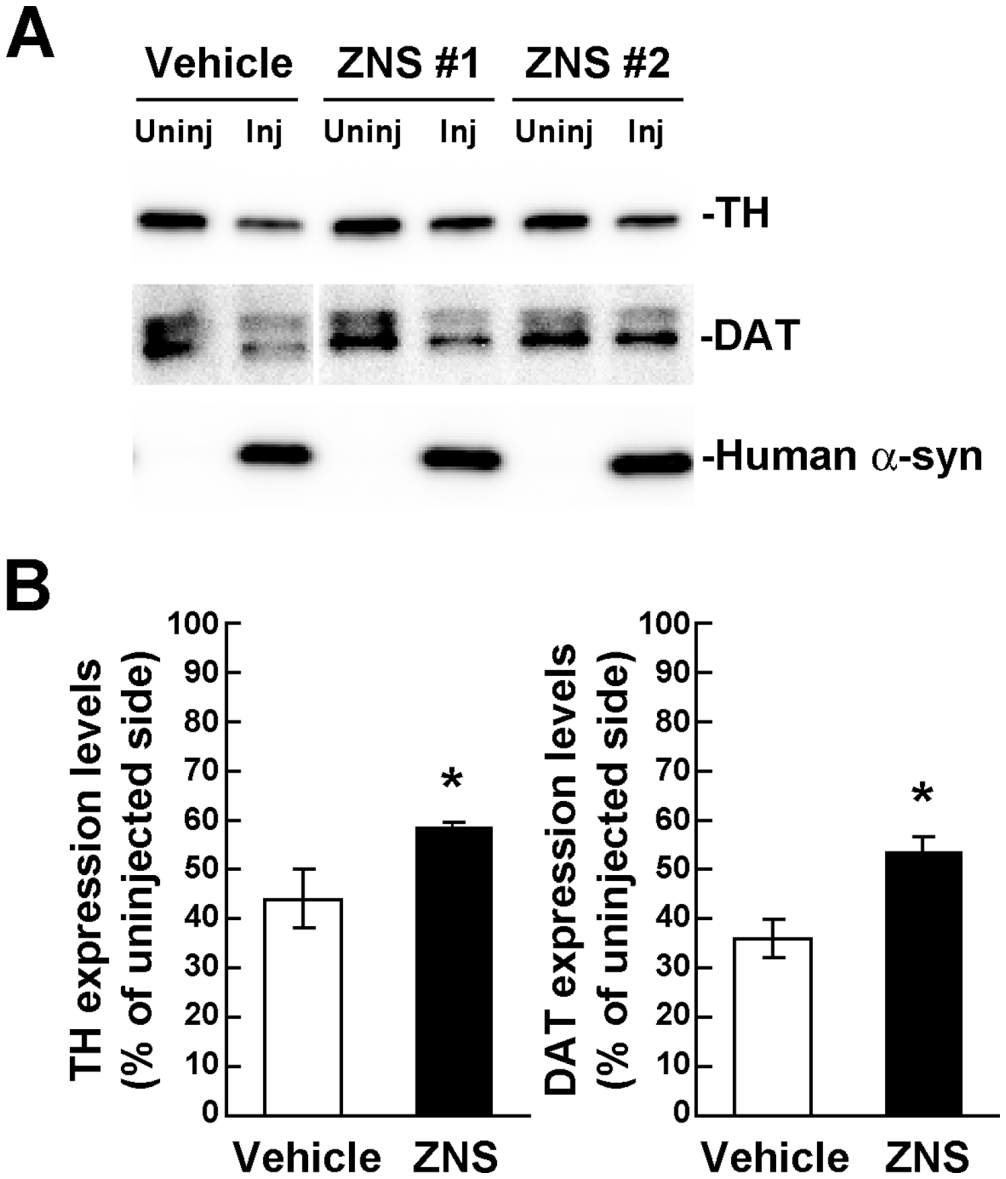
Biochemical analysis of the expression levels of TH and DAT in rats unilaterally injected with rAAV-A53T α-synuclein. The striatum tissues were collected at 4 weeks after viral injection, and divided into the injected and uninjected sides. (**A**) 1% Triton X-100-soluble extracts (10 µg/lane) were analyzed by western blotting with TH, DAT, and LB509 (human total α-synuclein) antibodies. Representative blot data of the vehicle and ZNS groups are shown. Human α-synuclein is observed only in the rAAV-injected sides. Although the DAT blot of vehicle-treated samples is separated from the blot of ZNS-treated ones, these blots are originally derived from the same blot. (**B**) Graphs show the ratios of TH and DAT expression levels in the ZNS group relative to those in the vehicle group. As compared with vehicle treatment, ZNS significantly retained the expression levels of TH (n = 4 for each group, *P* = 0.013) and DAT (n = 4 for each group, *P*<0.001). Data represent the means ± SD. *P*-values were estimated by unpaired *t* test (*P<0.05).

To confirm the immunohistochemical data, we biochemically analyzed the expression levels of TH and DAT in the rat striatum at 4 weeks after rAAV-A53T α-synuclein injection. In western blotting of 1% Triton X-100-soluble striatal extracts, the TH protein levels of the injected side were 44.0±5.8% of the uninjected side in the vehicle group (n = 4), whereas those of the injected side were 58.2±1.6% of the uninjected side in the ZNS group (n = 4) (Figure 4). The DAT protein levels of the injected sides were 36.8±3.9% and 53.5±3.0% of the uninjected sides in the vehicle and ZNS groups, respectively (n = 4 for each group) (Figure 4). Consistent with the immunohistochemical data, ZNS treatment significantly retained the expression levels of TH (*P* = 0.013 by *t* test) and DAT (*P*<0.001), as compared with vehicle treatment.

### Effects of Late Treatment with Zonisamide on A53T α-synuclein-induced Loss of Nigrostriatal Neurons

**Figure 5:**
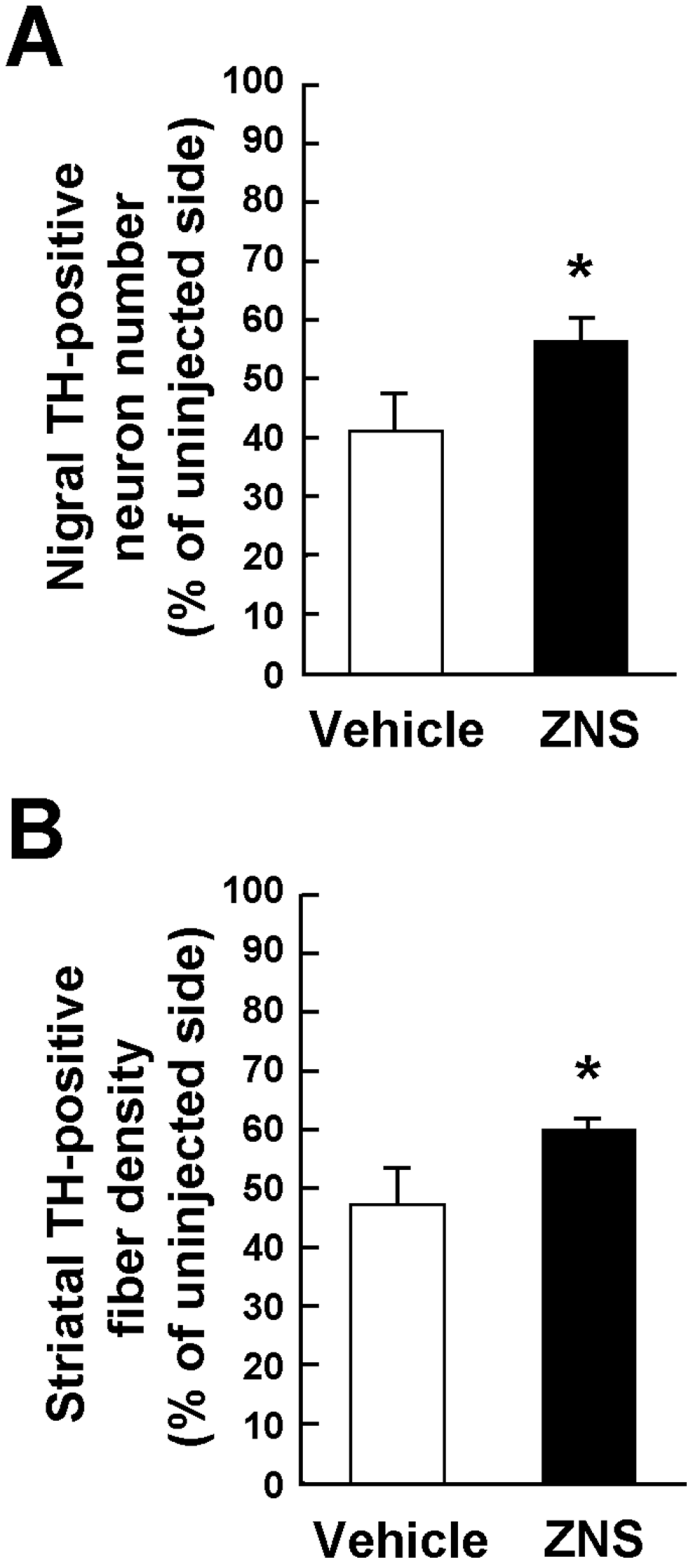
Effects of late treatment with ZNS on A53T α-synuclein-induced loss of nigrostriatal neurons. ZNS administration was started 7 days after rAAV-A53T α-synuclein injection, and the treatment was performed until 4 weeks after viral injection. Sections were immunostained with antibody for TH. Graphs show the quantitative analysis of the number of TH-positive nigral neurons (**A**) and the semiquantitative analysis of the optical density of TH-positive striatal fibers (**B**). As compared with vehicle treatment, ZNS significantly suppressed loss of TH-positive nigral neurons (*P* = 0.012). Decreased densities of TH-positive nigral fibers were significantly suppressed in the ZNS group (*P* = 0.020). Data represent the mean ± SD. *P*-values were estimated by unpaired *t* test (*P<0.05).

To further assess the protective effects of ZNS on A53T α-synuclein-induced loss of nigrostriatal neurons, we started administration of ZNS 7 days after rAAV-A53T α-synuclein injection, and treated rats until 4 weeks after viral injection. In the vehicle group, surviving TH-positive nigral neurons of the injected side were 40.9±6.6% of the uninjected side (n = 4) (Figure 5A). In the ZNS group, those of the injected side were 56.2±5.6% of the uninjected side (n = 4). Late ZNS treatment significantly attenuated loss of TH-positive nigral neurons (*P* = 0.012 by *t* test). Surviving TH-positive striatal fibers of the injected sides were 47.0±5.8% and 59.0±2.1% of the uninjected sides in the vehicle and ZNS groups, respectively (n = 4 for each group) (Figure 5B). Late ZNS treatment after viral injection also protected TH-positive striatal fibers from A53T α-synuclein-induced toxicity (*P* = 0.020 by *t* test).

### Effects of Zonisamide on A53T α-synuclein Aggregation

Five point mutations and multiplication of the *SNCA* gene, which encodes α-synuclein, are associated with rare families of autosomal dominant Parkinson’s disease [25]. It has been suggested that α-synuclein plays a causative role in the pathogenesis of both familial and sporadic Parkinson’s disease because fibrillar aggregates of α-synuclein, called Lewy bodies and Lewy neuritis, are commonly found as a pathological hallmark [26], [27]. Accumulating evidence shows that prefibrillar intermediates of α-synuclein, such as soluble oligomers and protofibrils, are highly toxic to neurons, whereas mature fibrils are less toxic [26]–[29]. The process of α-synuclein aggregation, eventually leading to the formation of Lewy bodies and Lewy neuritis, appears to be a major contributor to neurodegeneration in Parkinson’s disease [26], [27]. To elucidate whether ZNS exerted protective effects by affecting α-synuclein aggregation, we assessed the impact of ZNS treatment on the number of striatal aggregates containing Ser129-phosphorylated α-synuclein by a computer-based analysis. These aggregates were morphologically observed as axonal swellings and dystrophic terminals [15]. Ser129-phosphorylation of α-synuclein is a specific marker for detection of α-synuclein deposits [30]. Ser129-phosphorylated α-synuclein-positive aggregates are easily identified in the striatum because there is little immunoreactivity with an antibody for Ser129-phosphorylated α-synuclein in normal structures of the striatum, in contrast to an antibody for total α-synuclein, including phosphorylated and non-phosphorylated forms [15].

**Figure 6:**
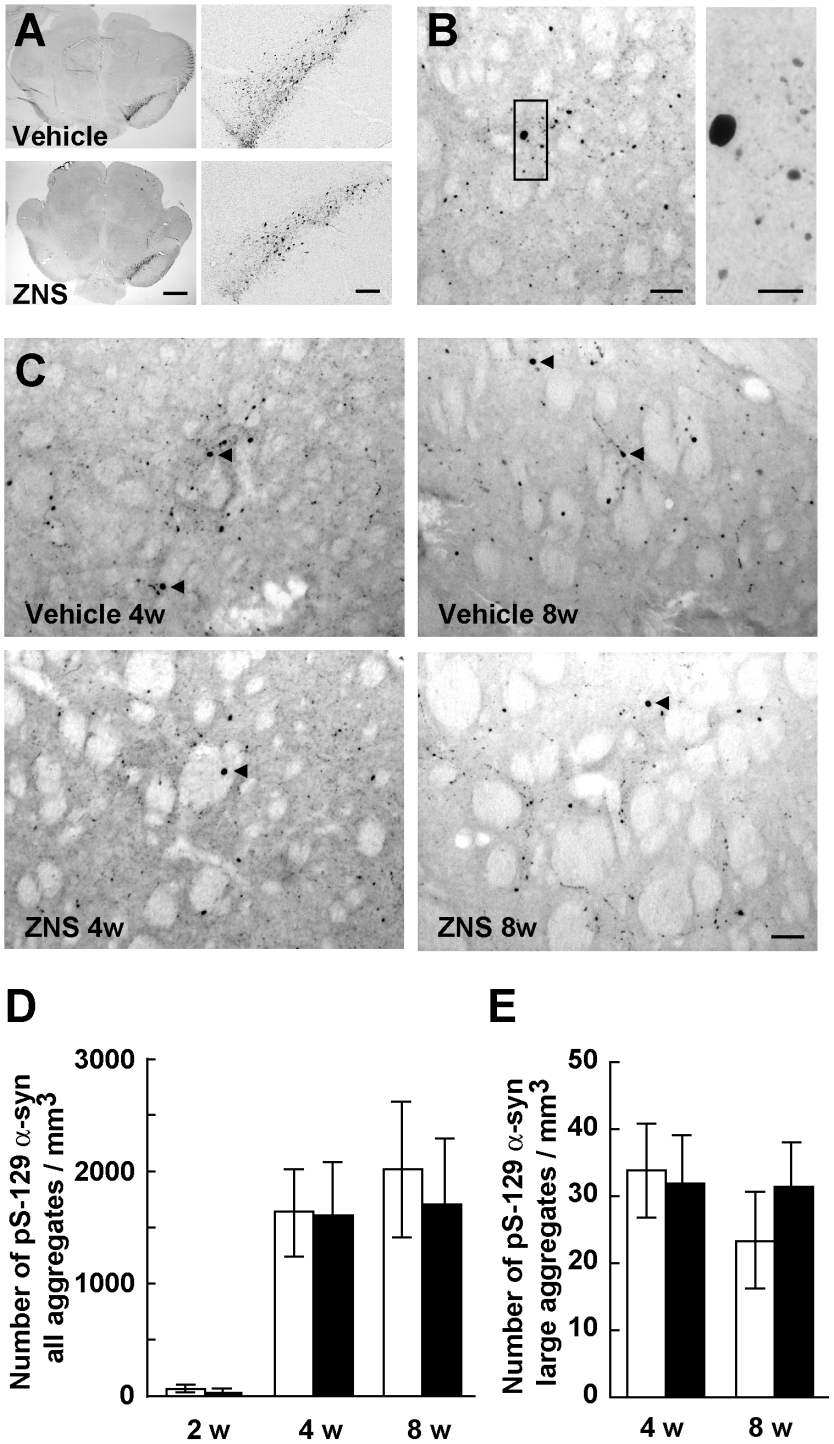
Analysis of Ser129-phosphorylated α-synuclein-positive aggregates in the striatum. (**A**–**C**) The sections were immunostained with antibody that specifically recognizes Ser129-phosphorylated α-synuclein. (**A**) Photomicrographs show representative immunostainings of the injected and uninjected sides of the substantia nigra at 2 weeks after rAAV-A53T α-synuclein injection. Left panels show images at low magnification, and right panels show enlarged images of the injected side of the substantia nigra. Scale bars indicate 1 mm and 200 µm in left and right panels, respectively. (**B**) High magnification photomicrographs show representative immunostainings of the injected sides of the striatum at 4 weeks after viral injection. Right photograph shows the enlarged image of the indicated area of left photograph. Scale bars, 50 µm (left panel) and 20 µm (right panel). (**C**) Photomicrographs show representative immunostainings of the injected sides of the striatum at 4 weeks (left panels) and 8 weeks (right panels) after viral injection in rats, which were treated with vehicle (upper panels) or ZNS (lower panels). Arrowheads indicate Ser129-phosphorylated α-synuclein-positive aggregates larger than 10 µm in diameter. Scale bars, 50 µm. (**D**) Graph shows quantitative analysis of all striatal Ser129-phosphorylated α-synuclein-positive aggregates, including small dot-like and large round structures, despite their size. Two-way ANOVA (group × time) demonstrated neither main effect of group [*F*(1,30) = 0.642, *P* = 0.429] nor significant group × time interaction [*F*(2,30) = 0.451, *P* = 0.641]. (**E**) Graph shows quantitative analysis of striatal Ser129-phosphorylated α-synuclein-positive aggregates larger than 10 µm in diameter. Two-way ANOVA (group × time) demonstrated neither main effect of group [*F*(1,20) = 1.195, *P* = 0.287] nor significant group × time interaction [*F*(1,20) = 3.202, *P* = 0.089]. The open bars illustrate the vehicle group and the black bars illustrate the ZNS group. Data represent the mean ± SD.

In the rats used in this assessment, ZNS administration was started 3 days before rAAV-A53T α-synuclein injection. As described above, at 2 weeks after viral injection, we found no significant difference in the expression levels of Ser129-phosphorylated α-synuclein in the midbrains between the vehicle and ZNS groups (Figure 1B, 6A). We first counted the number of all striatal Ser129-phosphorylated α-synuclein-positive aggregates, including small dot-like and large round structures, despite their size (Figure 6B). In the vehicle group, expression of A53T α-synuclein rapidly generated Ser129-phosphorylated α-synuclein-positive aggregates from 2 weeks (61.5±21.6/mm^3^, n = 6) to 4 weeks (1642.4±416.7/mm^3^, n = 6) after viral injection (Figure 6C, D). The number of striatal aggregates at 8 weeks after viral injection was 2014.1±608.1/mm^3^ (n = 6) (Figure 6C, D). These findings showed that the formation of striatal Ser129-phosphorylated α-synuclein-positive aggregates were correlated well with the progression of loss of nigrostriatal dopamine neurons. In the ZNS group, striatal aggregates were increased from 2 weeks (54.1±28.6/mm^3^, n = 6) to 4 weeks (1611.5±502.2/mm^3^, n = 6) after viral injection (Figure 6C, D). However, two-way ANOVA (group × time) demonstrated neither main effect of group [*F*(1,30) = 0.642, *P* = 0.429] nor significant group × time interaction [*F*(2,30) = 0.451, *P* = 0.641]. This indicated that there were no significant differences in the number of striatal aggregates between the vehicle and ZNS groups at 2 weeks and 4 weeks after viral injection (Figure 6C, D). In addition, at 8 weeks after viral injection, the number of striatal aggregates in the ZNS group (1702.6±587.9/mm^3^, n = 6) was comparable with that in the vehicle group (Figure 6C, D). To test whether ZNS affected the maturation of α-synuclein aggregates, we next investigated the number of striatal Ser129-phosphorylated α-synuclein-positive aggregates larger than 10 µm in diameter (Figure 6B, C). In the vehicle group, the formation of large Ser129-phosphorylated α-synuclein-positive aggregates was not observed at 2 weeks after viral injection. Striatal large aggregates appeared at 4 weeks (33.8±7.0/mm^3^, n = 6) and 8 weeks (23.4±7.1/mm^3^, n = 6) after viral injection (Figure 6C, E). In the ZNS group, large aggregates were found at 4 weeks (31.9±7.2/mm^3^, n = 6) and 8 weeks (31.5±6.2/mm^3^, n = 6) after viral injection (Figure 6C, E). Two-way ANOVA (group × time) demonstrated neither main effect of group [*F*(1,20) = 1.195, *P* = 0.287] nor significant group × time interaction [*F*(1,20) = 3.202, *P* = 0.089]. There were no significant differences in the number of striatal large aggregates between the vehicle and ZNS groups at 4 weeks and 8 weeks after viral injection (Figure 6C, E).

## Discussion

To elucidate whether ZNS has potential as a neuroprotective agent for the treatment of Parkinson’s disease, the present study focused on the effects of ZNS on α-synuclein-induced loss of nigrostriatal dopamine neurons and α-synuclein aggregation in a rat model, which expressed A53T α-synuclein using rAAV2. Expression of A53T α-synuclein caused drastic loss of nigrostriatal dopamine neurons from 2 weeks to 4 weeks after viral injection. ZNS treatment significantly delayed the pace of degeneration at 4 weeks after viral injection, and this protective effect lasted over 8 weeks of observation after viral injection. The formation of Ser129-phosphorylated α-synuclein-positive aggregates was clearly observed as Lewy neurite-like axonal swellings or dystrophic terminals in the striatum. These aggregates rapidly increased in number from 2 weeks to 4 weeks after viral injection. When we selectively assessed striatal Ser129-phosphorylated α-synuclein-positive aggregates larger than 10 µm in diameter, these aggregates similarly increased in number by 4 weeks after viral injection. These findings showed that the formation and maturation of Ser129-phosphorylated α-synuclein aggregates were closely correlated with the progression of neuronal loss. However, ZNS treatment failed to alter the total number of Ser129-phosphorylated α-synuclein-positive aggregates in the striatum as compared with that in the vehicle group. The number of these aggregates was almost constant at 4 weeks and 8 weeks after injection, although ZNS sustained the decelerating effect on neurodegeneration during this period. In addition, ZNS treatment did not affect the number of large aggregates (above 10 µm in diameter). These findings indicated that ZNS treatment delayed the pace of A53T α-synuclein-induced neurodegeneration, whereas striatal Ser129-phosphorylated α-synuclein aggregates were formed and matured at the similar level to vehicle treatment. This discrepancy suggests that ZNS neither blocks the toxic process of aggregation nor prompts the formation of mature non-toxic aggregates. It seems that ZNS administration prevents nigrostriatal dopaminergic neurodegeneration induced by A53T α-synuclein in a manner that is independent of α-synuclein aggregation. In our rat model of familial Parkinson’s disease, there may be an alternative pathway that is independent of the process of aggregation in α-synuclein toxicity, and ZNS may protect nigrostriatal dopamine neurons by modulating downstream process in cellular damage or a cell death pathway commonly caused by α-synuclein and neurotoxins.

MPTP exerts its toxicity toward dopamine neurons by inhibiting mitochondrial complex I activity, resulting in the production of toxic reactive oxygen species [5]. 6-OHDA damages dopamine neurons by generating reactive oxygen species, including hydrogen peroxide and derived hydroxyl radicals, via auto-oxidation and MAO-mediated deamination [5]. In addition, 6-OHDA has been known to interact with mitochondrial complex I and inhibit its activity [5], [31]. A previous study has shown that α-synuclein contains a cryptic mitochondrial targeting signal at its N-terminus, and it is partially localized to mitochondria in human dopaminergic primary neurons and in α-synuclein transgenic mice [32]. It has been reported that α-synuclein is predominantly associated with the inner mitochondrial membrane, where α-synuclein reduces mitochondrial complex I activity and increases production of reactive oxygen species [32]. These effects are enhanced by the A53T α-synuclein mutation [32]. In accordance with this study, Loeb et al. reported that overexpression of α-synuclein specifically inhibits mitochondrial complex I activity in A53T α-synuclein transgenic mouse brains [33]. These findings raise the possibility that MPTP, 6-OHDA, and α-synuclein commonly target mitochondrial complex I. Furthermore, they found that this inhibitory effect on mitochondrial complex I activity in transgenic mice is age-independent, and it is not associated with the formation of α-synuclein soluble oligomers and aggregates [33]. This finding may support the presence of an aggregation-independent pathway in α-synuclein toxicity. Mitochondrial complex I impairment causes the generation of reactive oxygen species [34]. The earlier article has demonstrated that ZNS has free radical scavenging activities by using electron spin resonance, and that ZNS exerts these activities in a dose-dependent manner at nanomolar concentrations [24]. These findings suggest that ZNS might protect neurons from α-synuclein-induced toxicity by suppressing the accumulation of reactive oxygen species caused by mitochondrial damage.

Neuroprotective therapies could target variable factors, such as oxidative stress, mitochondrial dysfunction, inflammation, protein aggregation, impaired clearance of misfolded proteins, and signals of apoptotic cascade, in the pathogenesis of Parkinson’s disease [4]. However, it remains unknown whether interference with any of these factors will be effective for a disease-modifying therapy [4]. To validate the protective effects of ZNS, it is necessary to elucidate whether there is an aggregation-independent pathway in α-synuclein toxicity, and the pathway is relevant to the neurodegeneration of Parkinson’s disease. The precise profiling of temporal correlation among mitochondrial complex I activity, α-synuclein pathology, and degeneration of dopamine neurons in animal models and Parkinson’s disease patients would provide important insights into these issues. Testing of ZNS in wild-type α-synuclein expressing animal models may help to evaluate its therapeutic potential in Parkinson’s disease. The present study assessed the effects of ZNS on nigrostriatal neurons by the pathological method. However, to accumulate the data on the protective effects of ZNS, it is also important to evaluate how ZNS has an impact on the behavior changes.
